# The Limits of Bayesian Thinking in Court

**DOI:** 10.1111/tops.12478

**Published:** 2019-10-31

**Authors:** Ronald Meester

**Affiliations:** ^1^ Department of Mathematics VU University Amsterdam

**Keywords:** Bayesian networks, Evidence, Likelihood ratios, Modeling, Legal case

## Abstract

We comment on the contributions of Dahlman and of Fenton et al., who both suggested a Bayesian approach to analyze the Simonshaven case. We argue that analyzing a full case with a Bayesian approach is not feasible, and that there are serious problems with assigning actual numbers to probabilities and priors. We also discuss the nature of Bayesian thinking in court, and the nature and interpretation of the likelihood ratio. In particular, we discuss what it could mean that a likelihood ratio is in some sense uncertain.

In this article, I comment on the contributions of Dahlman and of Fenton et al., who both suggested a Bayesian approach to analyze the Simonshaven case. The first part of Dahlman's contribution is general in nature and confirms that he is aware of some of the shortcomings and problems with the method at large. I agree with Dahlman that the Bayesian approach may be useful in avoiding the three problems he identifies: false dichotomy, dependence neglect, and miss rate neglect. In my experience, it is especially this latter problem that is a serious threat in many instances, and invoking Bayesian thinking can certainly be a safeguard against this. In fact, I find the *qualitative* message of Dahlman's paper quite convincing, and it is very well possible that the court indeed has overestimated the weight of the evidence in the Simonshaven case, as he suggests near the end of his contribution.

However, Dahlman does a lot more than that, and about the other, more quantitative part of his contribution I am much more critical. Roughly speaking, there are three main issues that I would like to discuss: (a) the numerical values of his likelihood ratios, (b) his interpretation of what Bayesian thinking *is*, and (c) the uncertainty and nature of a likelihood ratio. After I have discussed these issues one by one, I will move on to the contribution of Fenton et al. and comment on the (dis)advantages of their Bayesian network, compared to the linear Bayes approach of Dahlman.

*Numerical values of a likelihood ratio*. My greatest concern with Dahlman's paper is his way of assigning numerical values to likelihood ratios. It is hard to take these numbers seriously, and for most of his conditional probabilities, it would be hard to imagine that a well‐founded number can be assigned to it. Subjectivity does not mean that anything goes. The problem is that Dahlman gives no arguments for his choices. I believe that this is so because for many of his likelihood ratios, it would be impossible to assign a number in a well‐founded way. Invoking unfounded numbers seriously undermines the content of the message that Dahlman wants to convey, a message, I repeat, that I support to some extent. Take, for instance, the following quote:

At the end of the day it must be more likely that we would see a bundle of evidence if EL is guilty than if he is innocent. I estimate it as five times more likely. Likelihood ratio: 5.

Why 5? Why not 25? Why not 2? Such differences potentially matter a lot, especially (as is the case here) when many likelihood ratios must be multiplied.

It is in addition important to note that courts do not accept computations like the one given by Dahlman. In itself this is of course not a reason that they *should* not, but the arguments they give in their verdicts carry weight. In The Netherlands, reports containing Bayesian calculations covering a full case similar to the one of Dahlman have been submitted at least five times over the last few years, and in every single case, the court dismissed the Bayesian report. Let me quote from two of these verdicts. They can all be found on rechtspraak.nl, where one can search for the mentioned ECLI codes.

The court does not exclude that this method, if correctly applied, can be helpful in finding the truth, but the final answer to the question whether or not suspect can be convincingly proved, is not a question that can be answered by probability theory. (ECLI:NL:RBZWB:2016:3060, my translation)

However, the Bayesian method [ … ] has received a lot of criticism in the literature. [ … ] Besides it is doubtful whether or not probabilities of the components in the Bayesian method can be expresses in reliable numbers. The use of numbers creates an impression of objectivity. [ … ] Finally, it is questionable whether or not degrees of belief necessarily follow the axioms of probability theory. (ECLI:NL:GHSE:2018:421, my translation)

I think that this criticism is appropriate. In one case in which I have been involved myself, it turned out that the mathematics of a Bayesian analysis was inconsistent. Even when you interpret probability subjectively, as one should do in this context, there are rules that one should conform to in order to be sure that your mathematical model remains consistent. Just taking some unfounded numbers might very well lead to such inconsistency, especially when your mathematical model is vague or almost non‐existent. To illustrate what I mean by an inconsistency, it is, for instance, impossible to have events *A* and *B* so that *P*(*A*) = 0.2, *P*(*A*|*B*) = 0.8 and *P*(*A*|*B^c^*) = 0.5 are all true, where *B^c^* denotes the *complement*, or *negation* of the event *B*. Indeed, when the conditional probability of *A* given *B* and *B^c^* is, respectively, 0.8 and 0.5, then the *unconditional* probability of *A* must be between these two values, being a linear combination of them. This is a trivial example, but sometimes such inconsistencies enter the scene in a very subtle and non‐intended way, especially when conditional probabilities are derived from general statistics which are taken from different sources.

To be sure, I do not claim that Dahlman's computations contain inconsistencies, but I think Dahlman would have a hard time in defining precisely a mathematical model which is consistent with all his estimates, and which involves all events identified by him. And I do think that probability statements need such a model to be meaningful.

The last sentence in the quote above is also rather interesting. It refers to the fact that epistemic uncertainty does not always follow the logic of classical probability. For instance, it is possible that one has no belief in a particular statement but at the same time no belief in its negation either. If, for instance, a car caused an accident, and it is confirmed that there were two persons in the car at the moment of the accident, then it may happen that there is no information whatsoever about who was driving. In such a case, one's belief that person 1 was driving is small, but this does not imply that the belief that person 2 was driving is high. Hence, not all expressions of belief can be captured by probability theory. Epistemic uncertainty is, simply, more complicated than what probability theory allows for, and a more general setup for dealing with uncertainty might be called for; see, for instance, Cohen (1977), Kerkvliet and Meester (2017), Kerkvliet and Meester (2019), and Shafer (1976).

*What is Bayesian thinking?* Dahlman makes an interesting statement about the nature of the “Bayesian method”:

Critics of the Bayesian method say that it cannot mirror the complexity of the world. In my view, this misses the point of Bayesian thinking. Event trees and Bayesian Networks are not models of the world. They are models of our reasoning about the world, that help us avoid fallacies and biases.

How to assess this claim? First of all, I agree that no method whatsoever can mirror the complexity of the world, so the discussion should indeed not concern this point. But in his paper, Dahlman explicitly makes an attempt to model the situation around the Simonshaven case. This is, indeed, not an *objective* model of the world, but instead a subjective reconstruction that is by its very nature limited by lack of information. But even though it is not objective, it *is* a model. I do not think that an alleged model for our reasoning about the world would include black box numbers. Why would it, if only our *reasoning* is at stake? Black box numbers say nothing about reasoning. Instead, these numbers relate to our knowledge of the actual world, so I do not think that the claim of Dahlman stands in his own interpretation of the approach.

There are, of course, situations where explicit computations are meaningful, for instance when evaluating DNA evidence at the source level, and if that is the case, then these computations must be performed, and they will add to our understanding. But I see no reason why a court would have valued Dahlman's contribution differently than the examples that I mentioned above.

*The uncertainty of likelihood ratios*. Dahlman many times uses the word “estimate” when referring to likelihood ratios. He speaks about “subjective estimates with considerable uncertainty,” about “an approximate estimate of the miss rate,” and mentions that “the fact‐finder should therefore always remember that his/her result is just an approximation.” An approximation of what? These quotes strongly suggest that Dahlman has the impression that a likelihood ratio is an existing quantity that has to be approximated or estimated as if it were a model parameter. This, however, is a misunderstanding I believe.

First of all, setting up a likelihood ratio is entirely different than estimating a model parameter, such as the population frequency of a given DNA profile. This we can see by considering an extreme situation, namely a situation with no relevant data at all. In such a situation, obviously one cannot make a meaningful statistical estimate of any model parameter, and uncertainty remains. On the other hand, any likelihood ratio corresponding to such a situation is well defined and known, namely equal to 1. It is *precisely* equal to 1, without any uncertainty. Indeed, if the data give us no further information about two hypotheses *H*_1_ and *H*_2_, then the corresponding likelihood ratio must be equal to 1, and the posterior odds are equal to the prior odds. So the likelihood ratio must be of a different nature than a model parameter.

For example, consider a situation of a full match of a suspect *S* with a DNA profile found at the scene of the crime, with population frequency *p*. If this *p* were know to us, then the likelihood ratio of *S* being the donor versus an unrelated person being the donor would be 1/*p*. But in reality we do not know *p*, and we describe our knowledge about *p* by a probability distribution. When we do that, the likelihood ratio becomes a function of this probability distribution; that is, it is a function of our *knowledge* about *p*, and not of *p* itself.

If we make more observations, that is, if the data change, then so does the likelihood ratio, since our knowledge changes. The likelihood ratio is thus not a quantity that one could determine, or at least approximate arbitrarily well, if only enough data would be at our disposal. On the contrary, by its very nature, the likelihood ratio is a quantity that depends *only* on the data that we have seen (and our original conviction since one has to start somewhere). It is not some existing reality. There is simply no such thing as approximating a likelihood ratio “arbitrarily well” by performing more experiments. If we performed more experiments, the likelihood ratio would change, but it would be a *different* likelihood ratio depending on *different* data. It would not be a more precise version of the earlier likelihood ratio. Of course, if we would gather more and more data, we would become less uncertain in the sense that the acquisition of knowledge makes probability distributions on parameters converge to one with small variance. One may even imagine a situation in which no uncertainty exists at all anymore, and in which the corresponding likelihood ratio is exactly expressible in terms of model parameters, in the above case as 1/*p*. This will then be the appropriate likelihood ratio in such a situation, but it, simply, expresses something different than a likelihood ratio based on less data. In classical estimation procedures, the estimates always refer to the same underlying parameters, but in the case of likelihood ratios this is not true. They express conditional probabilities based on certain knowledge, and if the knowledge changes, so do the probabilities.

For a very detailed discussion of this issue, I refer to the forthcoming book (Meester & Slooten, 2020) and to the earlier discussion about this topic in Berger and Slooten (2016), Biedermann et al. (2016), Martire et al. (2016), Morrison (2016), Sjerps et al. (2015), Slooten and Berger (2017), Taylor et al. (2016), and VandenHout and Alberink (2016).

These considerations are certainly not only academic. They are fundamental, since they deal with what evidence *is*. Probability in this context can only be interpreted in a subjective way (Biedermann et al., 2016; Meester & Slooten, 2020; Taroni et al., 2018). Dahlman seems to agree with this, but he does not explicate what he calls the “uncertainty” of a likelihood ratio.

Moving on to the contribution of Fenton et al. (2016), we see that this contribution is of a completely different nature. One of the reasons people turn to Bayesian networks instead of a linear approach as the one of Dahlman, is that a Bayesian network is thought to have the capacity to deal with interdependencies. This is, in principle, true, but note that any Bayesian network can be reformulated in a linear Bayesian model, simply by adding one piece of evidence at a time. On the other hand, it is undeniable that the graphical structure helps assessing the dependencies, and at the very least the network automatically leads to a well‐defined mathematical model. This certainly is an advantage.

A Bayesian network is a graphical illustration of the joint distribution of a number of random variables. Designing such a joint distribution requires many choices. Two teams that would independently design a Bayesian network for the same case will undoubtedly end up with vastly different outcomes, and the present case is no exception. Also Dahlman has presented such a network (although he did not publish it) and indeed his network is very different from the one of Fenton et al. (2016). The conclusion must be that a complete description of a complicated situation in a legal case is subjective to the extent that numerical outcomes do not seem to mean very much. The sensitivity analysis performed by Fenton et al. does not really help, since it only deals with small perturbations of the current network. They say nothing about different networks and it may easily lead to the impression that the “truth” is close to what the network expresses. But this is not what the sensitivity analysis does for us.

The good thing with the approach of Fenton et al. is that it shows that certain canonical pieces can be identified, the so‐called *idioms*, that will play a role in many a network. But how these pieces are linked together, and which further nodes should be introduced, remains rather ad hoc.

The network in Fenton et al. is also interesting for another reason. It shows how difficult, if not impossible, it is to combine two competing narratives into one single network. What I mean by this is the following. Of the two nodes *Defendant killed her* and *Man in bushes killed her*, one, and only one, should be True (assuming that no other options are reasonable). But this is not automatically the case, of course. Given any prior probabilities for the relevant prior nodes, there is no reason in the world why their respective probabilities to be True will add up to 1. Conceptually it would have been much easier to merge the two nodes into one single node, taking two values denoting which of the two hypotheses is true. However, according to the authors (private communication), this would have led to a network with probability tables that become too large to execute. In Fenton et al. (2016), it is explained how this can in principle be solved by adding common descendants of the two nodes in question to make sure that one, and only one of the nodes, will be True. The naive solution by adding a single common child to condition on exactly one of the nodes to be True does not work, and a more complicated solution is necessary; see Fenton et al. (2016). However, this more complicated solution requires knowledge about the individual probabilities of the two nodes being True. If these individual probabilities were known, then the network was, after all, apparently not too large to execute, and the whole procedure of splitting the node would have been unnecessary.

The authors are, of course, aware of all this. From private communication, I understood that in the current case, they used the (incorrect) solution of adding a single node, but that they think that the error so introduced is not so significant. Whether this is true I cannot check, but incorporating various different narratives into one Bayesian network remains somewhat of a problem. In Neil et al. (2019), a different solution to this problem is proposed in the form of a Bayesian version of model selection, which essentially adds one layer to the Bayesian framework, in which each of the competing models has its own prior probability. This approach, however, does not solve my concern with the numerics, and introduces new ones, like another choice of priors.

There is one aspect that the contributions of Dahlman and Fenton at al. have in common: They both end up with posteriors, and therefore implicitly claim that they are in the position to assign numerical values to all necessary conditional probabilities and also to all priors. I find this remarkable since choices have to be made about issues that are not in the provenance of the forensic scientist or statistician who is designing the network. I am a mathematician, an expert in the use of probability and statistics in forensic science, but this does not mean that I consider myself able to actually provide numerical outcomes to all probabilities involved.

Exactly how useful the approaches of Dahlman and Fenton et al. are remains somewhat vague. I have serious concerns about this, essentially because I do not believe that one can reduce a complicated situation as the one in the Simonshaven case to a mere probabilistic calculation. There are all sorts of aspects that cannot be incorporated in probabilistic terms so easily, like the question *when* a suspect presents his or her alternative reading of the facts. Probability theory and statistics can certainly help in correctly assessing evidence, and can provide a safeguard against several potential mistakes. But making a full computation of a legal case stretches the applicability of probability theory much too far. In fact, I see a danger here: If Bayesian calculations get rejected all the time by the various courts, then this may jeopardize the acceptance of such calculations in those cases where they really make sense, and worse, they may convince legal representatives that Bayesian thinking is inappropriate and arbitrary. I see the first signs of exactly this happening right now in The Netherlands in current legal cases.

People often ask me what my alternative is, faced with my general criticism of the use of the Bayesian method. Expressing doubts about a certain method does not, of course, imply that a better one is available. I recall that I contested the outcome of a probabilistic calculation in the famous case against the nurse Lucia de Berk in The Netherlands. The reaction of the court was as follows: If you think this number does not make sense, give us a better number; see Meester et al. (2007). One should not try the impossible. In my own casework, I use a Bayesian approach primarily to aid the reasoning in the case. I use numbers only when these are both founded and relevant to the case. I help the judiciary with the logic of the case, and in my experience this is typically found very useful. As such, my own interpretation is perhaps close to, but not quite the same, as what Dahlman called a “model of our reasonings about the world, that help us avoid fallacies and biases.” Bayesian thinking has something to say, but please proceed with care and wisdom. It is always good to be aware of the limits of a certain theory, as this will make the useful applications of that theory only stronger.
